# ADAM10 Knockout from Human Glioblastoma and Colon Cancer Cells Modulates Diverse Signalling Networks and Inhibits Tumour Growth In Vivo

**DOI:** 10.3390/ijms262110684

**Published:** 2025-11-03

**Authors:** Hengkang Yan, Sakshi Arora, Linda Hii, Carmen Llerena, Mary E. Vail, Amr Allam, James R. W. Conway, Joel R. Steele, Han-Chung Lee, Ralf B. Schittenhelm, Andrew M. Scott, Peter W. Janes

**Affiliations:** 1 Olivia Newton-John Cancer Research Institute, Heidelberg, VIC 3084, Australia; 2 School of Cancer Medicine, La Trobe University, Heidelberg, VIC 3084, Australia; 3 Biomedical Discovery Institute, Monash University, Clayton, VIC 3800, Australia; 4 Department of Biochemistry and Developmental Biology, Faculty of Medicine, University of Helsinki, 00290 Helsinki, Finland; 5 Proteomics & Metabolomics Platform, Monash University, Clayton, VIC 3800, Australia

**Keywords:** ADAM metalloprotease, glioblastoma, tumour microenvironment, proteomics

## Abstract

ADAM10 is a transmembrane metalloprotease that regulates diverse signalling functions via the shedding of membrane protein ectodomains, and is implicated in tumour development, including glioblastoma multiforme (GBM) and gastrointestinal (GI) cancers, where high ADAM10 expression is associated with poor prognosis. We assessed the role of ADAM10 by gene knockout (KO) in U251 GBM cells, and its effects on protein shedding and protein expression on cell proliferation and on the growth of tumour xenografts in mice. The growth of tumours was severely delayed, relative to modest effects on proliferation in vitro, suggesting roles particularly in the context of the tumour microenvironment (TME). Proteomics analysis of KO cell-conditioned medium showed decreased levels of known ADAM10 targets such as Notch and Eph receptors and ligands, as well as other proteins involved in cell–cell adhesion, migration, signalling, metabolism, differentiation, and development, including angiogenesis. KO cell and tumour lysate analysis also showed modulation of proteins associated with metabolic and catalytic activity, cell–matrix organisation and differentiation. Similar effects were also observed in the SW620 colon cancer model, indicating broader significance. Furthermore, expression of the associated protein sets also correlated with ADAM10 expression in human GBM and colon cancer specimens (TCGA datasets), indicating clinical relevance. Collagens and proteins associated with matrix deposition and fibril organisation were notably reduced in ADAM10 KO GBM tumours, and histology confirmed decreased collagen fibrils and blood vessels. Unexpectedly, increased chondrocyte differentiation was evident in ADAM10 KO U251 tumours, suggesting a role for ADAM10 in maintaining an undifferentiated phenotype in vivo. Together, our data indicate the importance of ADAM10 in diverse signalling mechanisms in tumours and the TME that promote tumour development.

## 1. Introduction

ADAM (A Disintegrin and Metalloprotease) proteins constitute a large family of transmembrane proteins, with a complex extracellular region including disintegrin- and metalloprotease-like domains, although not all ADAMs are functional proteases [1]. ADAM10 and ADAM17 (also called TACE) are evolutionarily distinct members that are widely expressed and essential for normal development. They possess proteolytic activity and shed the extracellular domains of a range of adhesion molecules, cytokines, growth factors, and receptors, and are hence termed ‘sheddases’. While the two proteins share overlapping substrate specificity, ADAM10 has been shown to be essential for ligand-mediated Notch signalling [2], and ADAM10 knockout (KO) mice resemble the Notch knockout phenotype, with embryonic lethal defects in somite, neural, and vascular development [3]. ADAM10 cleaves both Notch ligands and receptors, facilitating the gamma-secretase-mediated release of the Notch intracellular domain (NICD), which controls gene transcription programmes associated with cell fate decisions in normal and tumour development, tissue homeostasis, and angiogenesis [4]. It also cleaves other developmental proteins, including Eph receptors and their membrane-bound ephrin ligands [5,6], and can cleave substrate cell-autonomously or across cell–cell junctions during juxtacrine signalling [6,7].

ADAM10 is reportedly over-expressed in various cancer types, where it is often associated with a more aggressive tumour phenotype and poor patient prognosis [8,9,10]. It was shown to be overexpressed in the aggressive brain tumour glioblastoma multiforme (GBM), associated with tumour-stage and stem cell-like characteristics [11,12,13,14], while TCGA mRNA expression data also showed upregulation in low grade glioma, linked to poorer overall survival [15]. ADAM10-mediated cleavage of N-cadherin was associated with decreased glioblastoma cell migration, and its cleavage of ligands for the NK cell receptor NKG2D was shown to reduce the immunogenicity of GBM and hepatocellular carcinoma cells [10,16,17]. ADAM10 has also been shown to function in tumour crosstalk with the GBM microenvironment (TME) [18], shedding the synaptic adhesion molecule neuroligin-3 (NLGN3) from neurons and oligodendrocyte precursor cells, which promotes GBM proliferation through the focal adhesion kinase (FAK) and PI3 kinase–mTOR pathways [19]. Other ADAM10-dependent signalling pathways are also implicated in GBM development, including Notch signalling [20,21], where ADAM10 mediates ligand-dependent Notch receptor activation, suggesting a more complex role in tumour development. ADAM10-mediated Notch signalling is also implicated in GI cancers [22,23], with roles in adenoma formation and stem cell maintenance, and antibodies targeting ADAM10 in colorectal (CRC) and GBM models inhibited tumour growth and impacted the tumour microenvironment [24,25,26,27]. To shed more light on ADAM10 function, we investigated the effects of ADAM10 deletion, primarily on human U251MG GBM cells in vitro and in mouse xenografts, assessing associated changes in the secretome, proteome, and tumour phenotypes. Key findings were also validated with the SW620 CRC model to assess significance across tumour types.

## 2. Results

### 2.1. Effect of ADAM10 KO on the Tumour Cell Secretome

**Figure 1:**
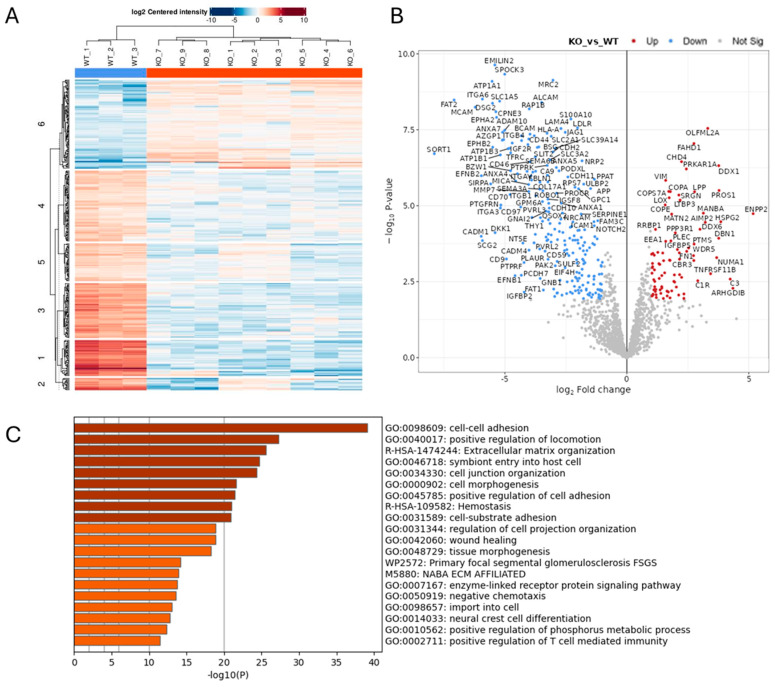
Proteomics analysis of conditioned medium from triplicate cultures of Wt U251 and three ADAM10 KO clones (clone 1: KO 1–3; clone 2: KO 4–6; clone 3: KO 7–9) (**A**,**B**). Heatmap (**A**), and volcano plot (**B**) of significantly, differentially expressed proteins (≥2-fold). (Blue: downregulated). (**C**) Gene ontology (GO)/protein annotation analysis of significantly downregulated proteins: bar graph shows enriched terms across input gene lists, coloured by *p*-value (https://metascape.org).

To investigate the function of ADAM10 in glioblastoma cells, we used CRISPR/Cas9 to delete ADAM10 from the human glioblastoma cell line U251MG (U251). We screened clones for genetic disruption by PCR analysis and DNA sequence analysis and confirmed loss of expression compared to parental wild-type cells (WT) by a Western blot of cell lysates (Figure S1). Since ADAM10 is an active sheddase, releasing the extracellular domain of membrane proteins, we compared the secretome of WT and ADAM10 KO U251 cells. To rule out non-specific changes due to clonal variation, we used three independent ADAM10 knockout (KO) clones, using label-free quantitative mass spectrometry of the conditioned medium from triplicate cell cultures. Principle component analysis (PCA, Figure S1C) of the 1729 quantifiable proteins detected showed highly reproducible replicates, with major differences between WT and KO clones, and only moderate inter-clonal variation. This contrast was also evident from a heatmap using unsupervised clustering, in which WT samples separated clearly from KO lines, which each clustered together (Figure 1A). Comparative analysis revealed 319 proteins with significantly altered expression levels (>2-fold) between WT and all combined KO samples (Figure 1B and Table S1). The majority of these proteins (224) showed a reduced expression upon KO of ADAM10 (Figure 1B and Table S1). This included proteins that were previously identified as ADAM10 substrates, such as Notch receptors and ligands (Notch2, JAG1), Eph receptors (EPHA2, A3, A5, B2) and their ephrin ligands (EFNB1, B2), and Cadherins (CDH2, 10, 11) (Figure S2). Interestingly, ADAM10 was also detected in the medium from the Wt U251 cells, possibly on exosomes, as previously reported [28], but not in KO samples (with only imputed values in KO samples), as expected.

Gene ontology (GO) analysis of downregulated proteins showed the significant enrichment of proteins involved in cell–cell and extracellular matrix interactions, cell motility, and morphogenesis or differentiation, including in neural and vascular development (Figure 1C and Figure S1D). In addition to Notch and Eph pathways, these include the desmosomal cadherin Desmoglein (DSG) 2 [29,30], as well as other cadherins (CDH2, 10, 11) and protocadherins (PCDH17, FAT1 and FAT2) [31], integrins (ITGA2, A3, A5, A6, B1, B4), and semaphorins (SEMA3A, 4B, 4D, 6B) (Figure S2). Receptor tyrosine kinase and MAP kinase signalling proteins were also downregulated in the ADAM10 KO medium, including the EGF/erbB1 receptor, erbB3/4 ligand NRG1, and MET [32]), and also the collagen-binding discoidin domain receptors DDR1 and DDR2 [33]. In addition to proteins involved in cell–cell and cell–matrix interactions, ADAM10 KO cell media showed decreases in proteins involved in vesicle trafficking and exosomes (e.g., sortilin Sort1), indicating likely effects not only on proteolytic shedding, but also endocytosis and exosome production. Immune regulatory pathways were also impacted, with the NK cell ligands MICA, MICB, and ULBP2 all being reduced in medium from ADAM10 KO U251 cells (Figure S2), in line with previous reports identifying these molecules as ADAM10 substrates [34]. Interestingly, several proteins upregulated in medium from ADAM10 KO cells shared similar functional annotations, including semaphorin SEMA3B and other ECM-related proteins (COL5A1, COL4A1, LOX, MATN2), suggesting possible compensation.

### 2.2. Effect of ADAM10 Inhibitor Treatment on the Tumour Cell Secretome

**Figure 2:**
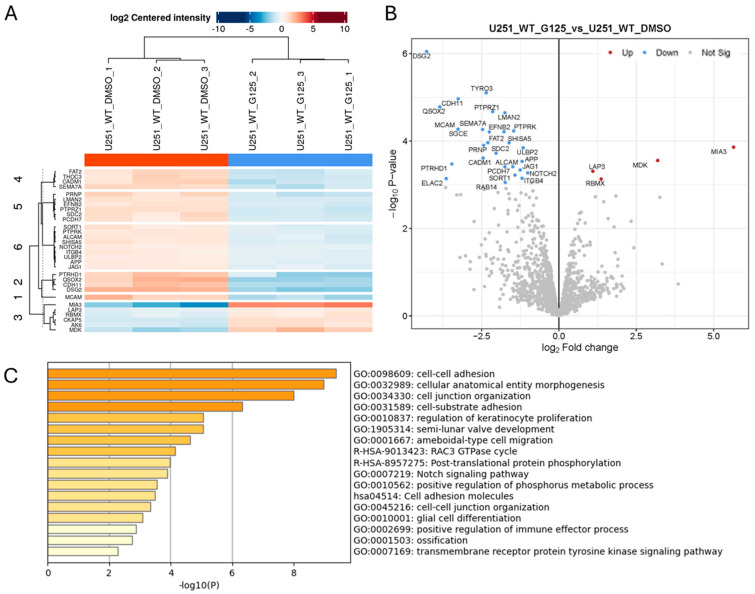
Proteomics analysis of conditioned medium from triplicate cultures of Wt U251 cells treated for 6 h with 10µM ADAM10 inhibitor GI254023X (G125) or DMSO vehicle control. Heatmap (**A**), and volcano plot (**B**) of significantly, differentially expressed proteins (≥2-fold). (Blue: downregulated in inhibitor-treated cultures). (**C**) Protein annotation analysis of significantly downregulated proteins: bar graph shows enriched terms across input gene lists, coloured by *p*-value (https://metascape.org).

Since the proteomics analysis of the conditioned medium from ADAM10 KO cells likely reflects the changes in direct proteolysis, but also the downstream effects, we also analysed the effects of short-term catalytic inhibition with the ADAM10 metalloprotease inhibitor, GI254023X. Cells were treated for 6 h with GI254023X (10µM) or a vehicle control, and the conditioned medium was recovered, concentrated, and analysed by label-free quantitative mass spectrometry, as above. This revealed a much smaller list of 27 proteins that were consistently and significantly downregulated in triplicate samples, and just 4 proteins were upregulated (Figure 2 and Table S2), from a total of 2773 quantifiable proteins. The lesser number of changes in proteins released into the medium might be expected from shorter term, partial inhibition of proteolytic activity, compared to gene knockout, where ADAM10 is totally ablated, and where longer-term effects on signalling and gene expression would also be more evident. Nonetheless, the majority of the proteins reduced after inhibitor treatment (19 out of 27) were common to those significantly reduced in KO cell media, indicating these proteins as likely to be direct ADAM10 substrates. In support, detected peptides were generally exclusively extracellular (including efnB2, JAG1, Notch2, DSG2, and QSOX2; Table S3). Similarly to ADAM10 KO, proteins downregulated by ADAM10 inhibitor treatment included examples of adhesion and signalling molecules associated with neuro- and vasculo-genesis (efnB2, DSG2, FAT2, ALCAM, MCAM, PCDH7, ITGB4, Notch2, JAG1), trafficking (SORT1), and immune responses (ULBP2). Known ADAM target DSG2 [29,30] and adhesion molecules cadherin CDH11 and MCAM were among the most highly downregulated proteins, as was the sulfhydryl oxidase QSOX2, which catalyses disulfide bond formation and isomerisation in extracellular proteins.

### 2.3. In Vitro and Functional Analysis of ADAM10 KO Tumour Cells

**Figure 3:**
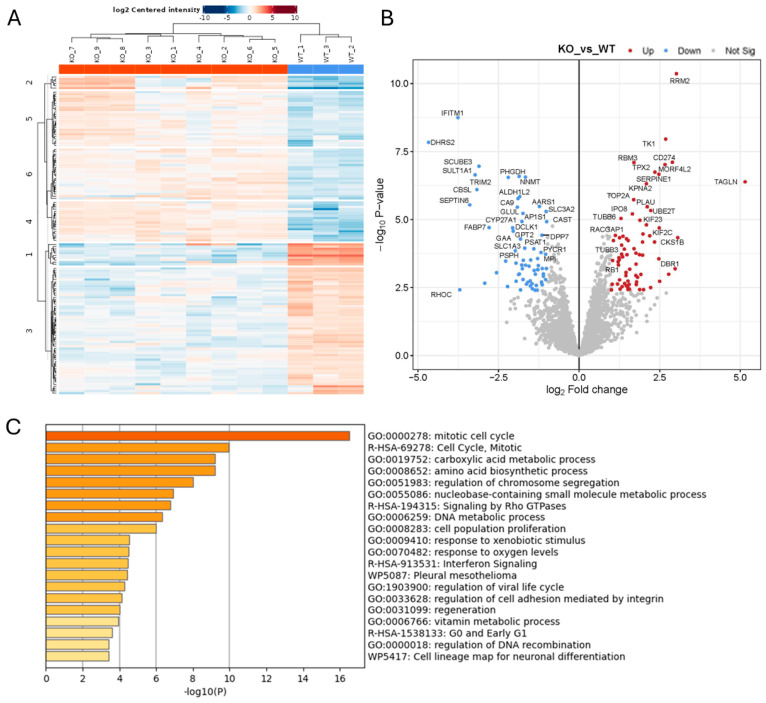
Proteomics analysis of protein lysates from Wt and ADAM10 KO U251 cells (**A**,**B**). Heatmap (**A**), and volcano plot (**B**) of significantly differentially expressed proteins (≥2-fold). (Blue: downregulated). (**C**) Protein annotation analysis of differentially expressed proteins: bar graph shows enriched terms across input gene lists, coloured by *p*-values (https://metascape.org).

We also performed proteomics analysis on cell lysates from WT and ADAM10 KO U251 cell lines, to assess more general impacts of ADAM10 KO on protein expression. A comparison of triplicate samples from WT and three KO clones combined showed 139 proteins with significantly altered expression (>2-fold) in all three clones, out of 3326 proteins that were reproducibly quantified (Figure 3 and Table S4). Only 20 of these overlapped with proteins altered in the conditioned media (Table S7, Figure S4), supporting the notion that changes in the secretome were predominantly a result of shedding changes, rather than overall changes in expression. In cell lysates, enrichment analysis showed significant changes in proteins involved in the cell cycle, metabolic and biosynthetic processes, and cell proliferation. Cell adhesion and Rho GTPase signalling pathways were also affected, consistent with effects on cytoskeletal function. Interestingly, extracellular domain peptides of the immune-checkpoint protein CD274/PD-L1 were more abundant in ADAM10 KO cell lysates, suggesting reduced shedding from ADAM10 KO cells, consistent with the previously reported correlation between soluble PD-L1 and ADAM10 expression [10,35], although the shed protein was not detected in the conditioned medium by mass spectrometry.

**Figure 4:**
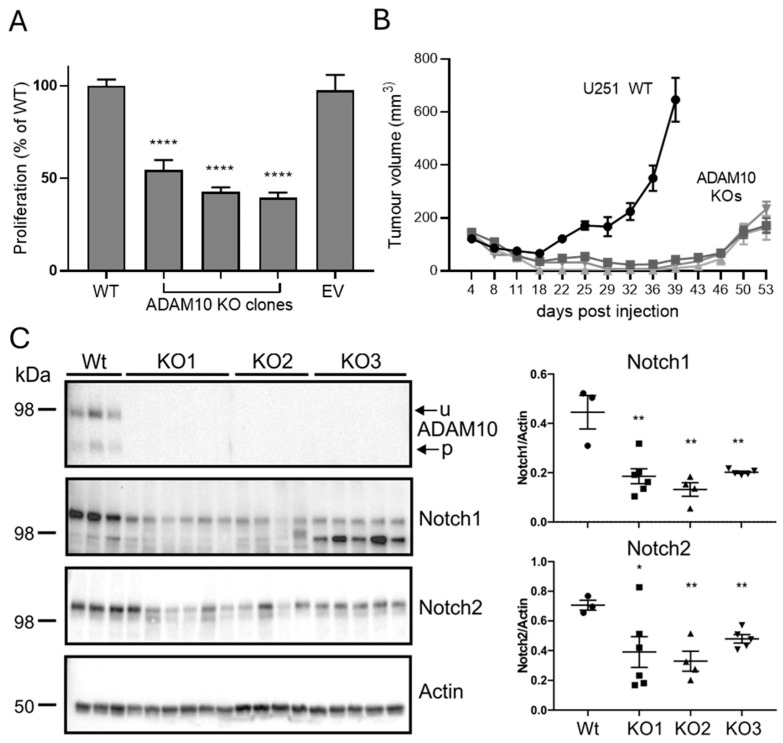
(**A**) Comparison of proliferation of Wt and ADAM10 KO U251 cells. Equal numbers of cells were plated in replicate wells and assayed after 3 days by MTS assay (mean ± SEM, *n* = 6, **** *p* < 0.0001). EV, control U251 cell line transfected with empty vector (Cas9 only). (**B**) Comparison of Wt and ADAM10 KO U251 clones, grown as subcutaneous tumours in NSG mice (mean volume ± SEM, *n* = 6/group). (**C**) Western blot of ADAM10 immunoprecipitates (top) or total tumour lysates, probed with antibodies for ADAM10 and Notch proteins, or actin as loading control. Graph shows quantification of band intensities, relative to actin (individual values with mean ± SEM; * *p* < 0.05, ** *p* < 0.01). u, unprocessed ADAM10; *p*, processed ADAM10.

To assess if these changes are reflective of the functional effects of ADAM10 loss, we analysed U251 ADAM10 Wt and KO cell lines by comparing cell proliferation/viability in vitro. We used MTS assays, which measure metabolic activity as a marker of proliferation, to compare equally seeded cultures in 96-well plates after 3 days. This showed a significant, approximately 2-fold reduction in the proliferation of all ADAM10 KO lines, compared to parental U251 cells or cells transfected with Cas9 alone (Figure 4A). This is in line with the changes in cell cycle and metabolic and biosynthetic pathways, noted above. We then assessed growth as tumour xenografts in immune-deficient mice, using subcutaneous tumours, as they are most amenable to replication and accurate determination of tumour volumes. In comparison to proliferation in vitro, growth of all ADAM10 KO U251 lines as tumours in mice was severely delayed, relative to wild type controls, being barely detectable after 6 weeks, when Wt tumours had begun to reach the ethical endpoint (Figure 4B). This suggested that ADAM10 function is particularly evident in the context of the tumour microenvironment (TME). We therefore recovered tumours taken at mid-stage (approximately 300 mm^2^), and prepared protein extracts for analysis (below).

### 2.4. Analysis of ADAM10 KO Tumour Xenografts

We firstly confirmed the ADAM10 KO in tumours by immunoprecipitation from tumour extracts, using the human ADAM10-specific antibody 4A11 [25] to differentiate ADAM10 in human tumour cells versus mouse cells of the host microenvironment. A Western blot of immunoprecipitates confirmed the absence of human ADAM10 from KO tumours (Figure 4C). As Notch signalling is a key pathway regulated by ADAM10, and we detected decreased shedding of Notch ligands and receptors after ADAM10 KO or inhibition in vitro, we also tested the levels of Notch in tumours by Western blot. Both Notch 1 and 2 were significantly decreased in KO, compared to Wt U251 tumours (Figure 4C).

**Figure 5:**
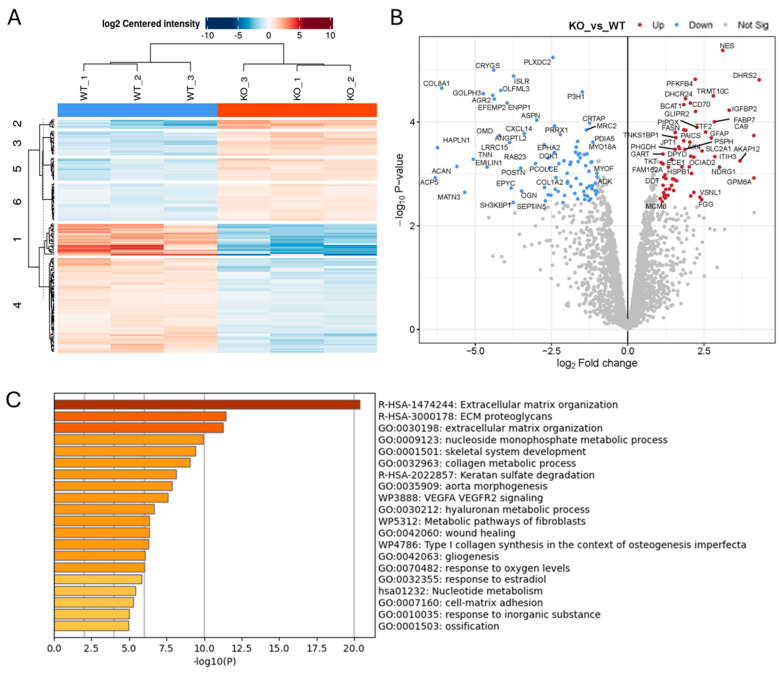
Proteomics analysis of protein lysates from Wt and ADAM10 KO U251 tumours, searched against the human protein database (**A**,**B**). Heatmap (**A**), and volcano plot (**B**) of all significantly differentially expressed proteins (≥2-fold). (Blue: downregulated). (**C**) Protein annotation analysis of significantly differentially expressed human proteins: bar graph shows enriched terms across input gene lists, coloured by *p*-values (https://metascape.org).

We also used label-free quantitative mass spectrometry to compare ADAM10 KO tumours to Wt U251 tumours taken at similar sizes (250–300 mm^3^), for unbiased identification of altered protein expression. Analysis against the human proteome for tumour cell-expressed proteins in triplicate biological replicates showed clear segregation of Wt or KO tumours on a heatmap plot, with 146 proteins out of 2751 quantifiable proteins having a significantly altered expression (>2-fold) (84 proteins significantly reduced in KO tumours, and 62 proteins increased) (Figure 5 and Table S5). Protein enrichment analysis of Wt versus KO tumours identified developmental processes as key functions, including blood vessel development, cell–cell adhesion and extracellular matrix organisation. There was some overlap with differentially expressed proteins from our previous analyses: 12 of the significant protein changes in tumours were common to those already identified in cell lysates, predominantly relating to metabolism, and 28 protein changes were in common with the identified secretome changes, including proteins related to ECM organisation and cell adhesion (Supplementary Figure S4). However, the majority of significantly differentially expressed proteins in KO tumours were unique to tumours, underlining the impact of the tumour microenvironment on ADAM10 function. Proteins downregulated in KO tumours with roles in regulating adhesion and angiogenesis included EphA2, integrin ITGB5, MCAM, and MYLK (myosin light chain kinase). Several collagens were also downregulated, as well as the collagen cross-linker LOXL3, angiopoietin-like protein 2 (Angptl2), which is associated with collagen-rich matrix and fibrosis [36] and aggrecan (ACAN), associated with collagen fibril organisation and cartilage development. Interestingly, other proteins associated with cartilage/chondrocyte development were also modulated (eg. BGN, OGN, MATN3, EPYC, ANXA6) (Figure 5 and Figure S5). Analysis against the mouse protein database, for changes in the host mouse TME, identified 43 proteins that were significantly altered (Figure S3 and Table S6), of which 30 were shared with the human protein search, and therefore could originate from either tumour cells or the TME. Amongst these, ECM and adhesion proteins were again prominent, including ACAN, OGN, HAPLN1, OLFML3, and the integrin-binding Tenascin N. Several ECM-associated proteins were only identified in the mouse protein search, such as multiple fibrinogens, Cartilage Oligomeric Matrix Protein (Comp), and Cathepsin K, indicating that KO of ADAM10 from tumour cells modulates the TME cell types contributing to matrix remodelling, particularly related to stromal fibroblasts.

**Figure 6:**
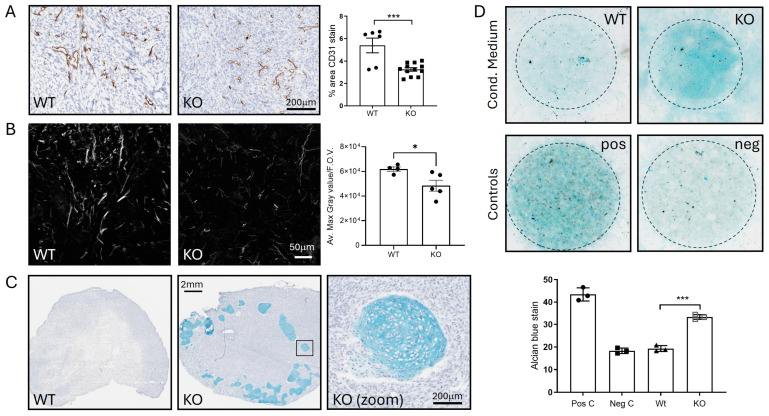
(**A**–**C**) Analysis of Wt and ADAM10 KO U251 tumour xenografts. (**A**) IHC with anti-CD31 staining of blood vessels. Graph shows % positive pixels in whole sections of n = 6 Wt and n = 15 KO tumours (mixed clones). (**B**) Visualisation of collagen by SHG, using 2-photon confocal microscopy. Graph shows quantitation of max. grey value/field of view (F.O.V.) averaged from 4 FOVs/tumour (4 Wt, 5 KO). (**C**) Alcian blue staining of tumours, showing chondrocytes in ADAM10 KO tumours, with zoom on boxed region. (**D**) Chondrocyte differentiation assay—MSCs grown as colonies (circled) in conditioned medium from Wt and ADAM10 KO U251 cells stained with Alcian blue. * *p* < 0.05, *** *p* < 0.001. Neg C (negative control)—medium only; Pos C (positive control)—chondrocyte differentiation medium.

We then analysed tumours for histological changes associated with the above changes in protein abundance. We analysed blood vessel density by staining for the endothelial marker CD31, since along with Notch, we had identified reduced shedding of several proteins linked to angiogenesis. Vessel staining was significantly reduced in ADAM10 KO U251 tumours, compared to Wt (Figure 6A). We also analysed the tumour ECM, particularly collagen fibrils, by second harmonic generation (SHG) imaging. This indicated a decrease in fibrils in ADAM10 KO tumours, reflecting the reduced levels of collagens, the cross-linker LOXL3, and other ECM proteins identified above (Figure 6B). Unexpectedly, we also noted clumps of cells with a chondrocyte-like morphology in ADAM10 KO, but not Wt U251 tumours. These stained positively with Alcian blue, a dye that binds to polysaccharides in cartilaginous ECM, supporting their chondrocytic identity (Figure 6C). Interestingly, several fibrinogens were notably more abundant in ADAM10 KO tumours and are known to be associated with chondrogenic differentiation [37]. Since the alterations of proteins associated with chondrocyte development were evident in tumours and not cultured cells, and included mouse proteins, this indicated possible involvement of the TME: specifically, stem-like stromal progenitor cells (mesenchymal stromal/stem cells, or MSCs) that can differentiate into chondrocytes. We therefore tested if conditioned medium from parental or ADAM10 KO U251 tumour cells could initiate differentiation of mouse MSCs in culture. Indeed, MSCs grown in conditioned medium from ADAM10 KO but not Wt U251 cells showed increased staining by Alcian blue, indicative of chondrogenic differentiation (Figure 6D). Since only the ADAM10 KO tumours showed chondrocyte differentiation, and only the ADAM10 KO cell-conditioned medium caused MSC chondrocyte differentiation, this suggests that the loss of ADAM10 from U251 cells leads to changes in secreted factors that inhibit cell stemness and promote differentiation.

To determine if ADAM10 plays a role in modulating similar pathways in other tumour types, we also used CRISPR/Cas9-deletion of ADAM10 in the SW620 CRC cell line, since, as described above, ADAM10 is upregulated in GI cancers, associated with stem-cell maintenance, and SW620 cells share stem cell-like characteristics with U251 cells [38,39]. Proteomics analysis of the conditioned medium again showed a range of proteins with altered expression (Figure S6A), and 39 proteins were also identified as being regulated in the secretome of ADAM10 KO U251 cell lines (Table S8). Shared downregulated proteins included ADAM10, SORT1, CADM1, CADM4, ALCAM, QSOX2, TMSB4X, PCDH7, and stem cell markers EPHB2 and CD46. Accordingly, similar functional pathways were impacted, including cell–cell adhesion, ECM/matrisome regulation, angiogenesis, protein phosphorylation, and innate immune cell function (eg. neutrophil granulation) (Figure S6B). Growth of ADAM10 KO SW620 cells as tumours in mice was also significantly slower, compared to parental WT cells (Figure S6C). Tumours also displayed significantly less staining for CD31+ endothelial cells, and the stem cell marker CD133, consistent with ADAM10 promoting a more stem-like, poorly differentiated phenotype (Figure S6D,E). Western blotting of anti-human ADAM10 immunoprecipitates from tumours confirmed the loss of hADAM10 expression in KO tumour cells (Figure S6F).

Lastly, to relate our findings to human tumours, we used the TCGA database to compare ADAM10 expression in human GBM to the expression of the protein sets we identified as being modulated by ADAM10. Indeed, all the identified protein signatures were found to correlate with ADAM10 expression in clinical samples (R values 0.52 to 0.66, Figure S7A). Similarly, we identified that key GO functional gene sets were also correlated with ADAM10 expression in human tumours, including cell–cell interaction, Notch signalling, angiogenesis, ECM organisation, morphogenesis, and phosphate metabolism (Figure S7B). This supports the relevance of our data for clinical GBM. Also, the proteins that were identified as being significantly regulated in the secretomes of both U251 and SW620 cells also correlated with ADAM10 expression in the TCGA datasets of both GBM and CRC tumours (Figure S8), indicating a likely clinical significance across multiple tumour types. Together, our results indicate that ADAM10 regulates tumour development via diverse signalling pathways, promoting ECM reorganisation, tumour angiogenesis, and a more fibrotic phenotype.

## 3. Discussion

Expression of ADAM10 has been reported to be upregulated in glioblastoma, and to correlate with a higher tumour grade [11,12], although conflicting data have been reported. Analysis of TCGA data showed elevated expression of ADAM10 in both GBM and low-grade glioma (LGG), relative to normal tissue, correlating with poor prognosis in LGG and also suggesting a role in early disease [15]. Previous studies have shown putative roles of ADAM10 in a variety of functions, including promoting cell stemness, migration and invasion in vitro [14], modulating immunogenicity [34,35,40], and mediating tumour-microenvironment cross-talk via shedding of the synaptic adhesion molecule neuroligin-3 (NLGN3) from neuronal cells [19]. These studies have generally employed ADAM10 inhibitors, which can have limitations in terms of efficacy and specificity, with even the most specific inhibitors also impacting other ADAMs and MMPs [41,42,43]. We therefore used a genetic knockout of ADAM10, from U251 GBM cells by CRISPR/Cas9, to investigate the resulting changes in protein shedding, protein expression, the proliferation of cells in vitro, and tumour xenografts in mice.

A comparison of the conditioned medium from parental and ADAM10 KO U251 cells showed a decrease in a large number of proteins, particularly those related to the cell-surface receptor signalling associated with development. These included adhesion molecules (cadherins, integrins, DDRs), tyrosine kinase receptors and other receptors controlling cell guidance (Eph/ephrins, MET, semaphorins), and cell fate/stemness (Notch, Wnt pathway). NK cell ligands (MICA, MICB, ULBP2) were also downregulated, and are previously reported substrates [34]. Use of the ADAM10-selective inhibitor identified far fewer proteins, but included examples of most of these groups as evidence of them likely being direct targets of ADAM10-mediated shedding. Interestingly, the single-pass membrane protein, QSOX2, was consistently identified in these experiments and was previously reported to be reduced in the medium from ADAM10 KO neurons [44]. QSOX2 was found to regulate the sensitivity of neuroblastoma cells to interferon-gamma-induced apoptosis, also suggesting a role in immune regulation in GBM. It was also reduced in our proteomic analysis of the secretome of ADAM10 KO SW620 cells, along with a number of other protein changes that are common to both tumour models, suggesting broader tumour relevance. As QSOX2 catalyses disulfide bond isomerisation, which is thought to mediate conformational change in ADAMs that regulate their activity [45,46,47], this suggests that there may be two-way regulation of QSOX2 and ADAM10.

Analysis of lysates from ADAM10 KO and WT U251 cells revealed changes consistent with those identified above, including downregulation of stem cell markers, such as DCLK1 and ALDH1L2, in keeping with the effects on Notch and Wnt pathway members, and the increased differentiation noted in tumours. Indeed, knockout of ALDH1L2 from U251 cells has previously been shown to slow growth and promote differentiation [48]. Stem cell markers such as EPHB2, CD46, and CD133 were also notably reduced in the ADAM10 KO SW620 model. We also noted changes in cell metabolism and cell cycle proteins, again consistent with reduced ‘stemness’. RhoC, which links membrane receptors with focal adhesions and actin/myosin networks and is associated with cancer cell stemness, migration, and angiogenesis [49], was downregulated, and many other proteins that were associated with cell cycling were also modulated.

Similarly, metabolic changes were also evident in tumours. Carbonic anhydrase IX (CA9) is a transmembrane enzyme, identified as being downregulated in the ADAM10 KO cell-conditioned medium and cell lysates, and was previously identified as a substrate for ADAM10 [50]. Interestingly, CA9 was upregulated in tumours, suggesting possible adaptation in vivo, consistent with its known roles in regulating pH during tumour adaptation to hypoxia and acidosis, facilitating metabolic reprogramming of cancer cells. Other prominent changes were in proteins associated with extracellular matrix (ECM) organisation, and in particular the formation of collagen fibrils, with multiple collagens, integrins, the collagen cross-linker LOXL3, and the integrin-binding tenascin all reduced. Indeed, collagen fibrils were less evident in ADAM10 KO tumours by second-harmonic generation (SHG) microscopy. While use of a subcutaneous model has potential limitations compared to organ-matched orthotopic models, our results are consistent with recently reported roles for ADAM10 in fibrosis in different orthotopic models [51,52]. Since these ECM proteins were modulated primarily in the context of tumours and the tumour microenvironment, and ECM deposition and fibrosis are particularly associated with cancer-associated fibroblasts (CAFs), this suggests a role for ADAM10 in modulating tumour–fibroblast crosstalk. Wnt signalling, which we identified as downregulated, is required for fibroblast activation, and LRRC15, a marker for activated, myofibroblast-like CAFs [53], was also notably downregulated in KO tumours. These CAFs drive fibrosis, which has also been reported to depend on Notch shedding and activation [54].

Fibroblasts/mesenchymal cells in the TME may also contribute to the unexpected chondroid phenotype we observed in ADAM10 KO tumours. The presence of cartilage and chondrocytes has been reported previously in glioma and generally ascribed to chondroid metaplasia in the mesenchymal stroma, although transitions from astrocytes to chondroid cells have also been reported, characterised by the increasing deposition of chondroid ECM [55]. In support, glioma stem cells were reported to undergo mesenchymal differentiation in vitro and as xenografts in mice, producing osteo-chondrogenic areas, and associated with reduced proliferation [56]. Notch signalling is implicated in chondrocyte development, where increased active Notch resulted in decreased cartilage precursor proliferation and inhibited chondrocyte differentiation [57]. Consistent with this, our results show decreased Notch and increased chondrogenesis in ADAM10 KO tumours, in keeping with a more differentiated and less stem-like tumour phenotype.

Finally, the correlation of the identified ADAM10-dependent protein expression changes with the ADAM10 expression in human GBM and CRC datasets indicate the relevance of our data for human disease. Key GO functional protein sets that were modulated by ADAM10 also correlated with ADAM10 expression in human tumours, including proteins involved in cell–cell interaction, Notch signalling, angiogenesis, and ECM organisation. Together, our data thus support therapeutic targeting of ADAM10 in GBM. Furthermore, the changes we saw in the expression of proteins regulating both innate and adaptive immunity, including NK cell ligands and the immune-checkpoint protein PD-L1, suggest the further possibility of synergy with immunotherapy. A limitation of this present study is the use of subcutaneous xenografts in immunodeficient mice, and further investigation of immune-mediated effects could be undertaken in future, utilising immune-competent mice and orthotopic models.

## 4. Materials and Methods

### 4.1. Cell Lines, Maintenance, and Proliferation Assay

The U251MG (U251) and SW620 cell lines were from ATCC and maintained at 37 °C and 5% CO_2_ in Dulbecco’s Modified Eagle Medium Nutrient Mixture F-12 [DMEM/F-12, containing L-Glutamine and sodium bicarbonate (2.438 g/L)] (Gibco, Scoresby, VIC, Australia), and supplemented with 10% Foetal bovine serum (FBS) (Moregate Biotech, Bulimba, QLD, Australia) and 1% penicillin-streptomycin (10,000 U/mL penicillin; 10 mg/mL streptomycin) (Gibco). Cells were passaged at 1:10 when they reached approximately 80% confluence, using TrypLE^TM^ Express (Gibco) for detachment. Proliferation assays were performed by plating 1000 cells/well in 96-well plates, in DMEM with 2% FCS, and viable cells were measured using an MTS assay (CellTiter 96, Promega, Madison, WI, USA) after 3 days.

### 4.2. Generation of ADAM10 Knockout Cell Lines

The following guide RNAs (gRNAs) were cloned in the pLentiCrisprV2 vector (Genscript): 5′ GATACCTCTCATATTTACAC; 5′ AAGTGTCCCTCTTCATTCGT. We used two gRNAs to avoid off-target effects. A total of 10 µg of each gRNA and 3 µg of pSpCas9-px165 were electroporated into 10^7^ U251-MG cells. The electroporated cells were resuspended in 10 mL of DMEM media and transferred into 10 cm cell culture dishes (Corning, Corning, NY, USA) for 2 days, before 2 µg/mL puromycin was added to select for transfected cells. Colonies were picked and transferred into 96-well plates, then passaged into duplicate 24-well plates. DNA extracts from clones were screened by PCR (Forward Primer: GGGTTCTGTTAAGGTCCAACT, Reverse Primer: AGAGTCCTACCACAGCTACTAA). DNA was isolated from cells lysed in 300 µL 50 mM Tris-HCl pH8.0, 100 mM EDTA, 100 mM NaCl, 1% sodium dodecyl sulphate; protein was precipitated with 100 µL 10 M ammonium acetate and cleared by centrifugation; DNA was precipitated from supernatant with 400 µL of propan-2-ol, followed by centrifugation. Pellets were washed in 70% ethanol, dried, and dissolved in 25 µL Tris-EDTA buffer.

### 4.3. Mass Spectrometry Experimental Design and Data Analysis

Sample Preparation: For conditioned medium, triplicate 15 cm dishes of cells at 90% confluence were washed with serum free media three times, then incubated in 25 mL serum free media for 6 h, which was recovered and centrifuged at 4000× *g* for 5 min to remove insoluble material, followed by filtration and concentration to 300 µL per sample, using Amicon Ultra Centrifugal Filter units 10 kDa (Merck Millipore, Burlington, MA, USA). Protein concentrations were measured using a BCA kit (Thermo Fisher Scientific, Scoresby, VIC, Australia) and normalised amounts of protein were reduced and alkylated with 10 mM TCEP (Thermo Fisher Scientific) and 40 mM chloroacetamide (Merck Millipore) with incubation at 55 °C for 15 min. Enzymatic digestion was performed using Trypsin Gold (Promega, Madison, WI, USA) at a 1:50 wt/wt ratio overnight at 37 °C.

For cell and tumour samples, cell pellets (10 cm dish) or tumours (50 µg chunks) were solubilised in 5% SDS, 100 mM Tris-HCl, and heated at 95 °C for 10 min to denature enzymes. DNA was sheared using ultra-probe sonication (Qsonica, Newtown, CT, USA, Q125) in three 30 s intervals with resting periods on wet ice. Insoluble debris was removed by centrifugation, and the supernatant was processed using the S-trap protocol, according to the manufacturer’s instructions [58]. All peptide samples were cleaned up by solid phase extraction on C18 ZIP tips for secretomes and stage-tips packed with SDB-RPS (Merck Millipore) [59] for tumour samples. All samples were acidified with 0.1% formic acid upon reconstitution and spiked with iRT peptides.

Mass Spectrometry Acquisition: Chromatographic separation was conducted using a Dionex Ultimate 3000 RSLCnano system, which was coupled to either a QExactive Classic or an Orbitrap Fusion mass spectrometer (Thermo Fischer Scientific). Tryptic peptides were separated by increasing concentrations of 80% acetonitrile (ACN)/0.1% formic acid at a flow of 250 nL/min on an Acclaim PepMap RSLC analytical column (75 µm × 50 cm, C18, 2 µm, 100 Å), with a trap column (Acclaim PepMap 100, 100 µm × 2 cm, C18, 5 µm, 100 Å). The mass spectrometers operated in data-dependent acquisition mode using in-house, label-free quantification (LFQ)-optimised parameters, with 120 min of separation used for each sample. This was performed by the Monash Proteomics and Metabolomics Facility.

Data Analysis: For secretomes of Wt or ADAM10 KO cells, label-free quantification (LFQ) was performed using MaxQuant v1.6.2.10 [60] with the Andromeda search engine, applying a 1% FDR cut-off. The human SwissProt database was utilised for all analyses, with carbamidomethylation as a fixed modification and oxidation of methionine and acetylation of the protein N-terminus as variable modifications. Statistical analyses were conducted in Perseus v1.6.2.3, with protein quantification based on log2-transformed LFQ intensities and significant fold-changes determined using a two-sided T-test with error-corrected *p*-values.

For all other experiments, protein identification and quantification were performed using Fragpipe with MSFragger [61] and a fast deisotoping algorithm, and its implementation in the MSFragger search engine [62] as the search engine against the human or mouse SwissProt Databases, is indicated. The standard label-free quantification match-between-runs (LFQ-MBR) workflow was applied with no changes to workflow, employing IonQuant [63] and the MaxLFQ method of protein abundance calculations. A percolator was used for PSM validation, with strict Trypsin as the enzyme, allowing up to two missed cleavages. A 1% FDR cutoff was applied at the protein level. Statistical analysis and visualisation were performed using standard settings in LFQ-Analyst from the Monash Proteomic Analyst Suites (https://analyst-suites.org/, accessed on 8 May 2025) [64,65].

Protein list pathway analysis was performed with Metascape (https://metascape.org, accessed on 8 May 2025), using significant, ≥2-fold changes in triplicate samples. Cytoscape (version 3.10.1; https://cytoscape.org) was used to generate network plots using a subset of enriched terms (no more than 15 terms per cluster and no more than 250 terms in total), where each node represents an enriched term and is coloured by its cluster ID, and terms with a similarity of >0.3 are connected by edges.

### 4.4. Protein Extracts for Immunoprecipitation and Immuno-Blotting

Cells were lysed in 1% Triton X100 in KALB lysis buffer (50 mM HEPES, 5 mM EDTA, 150 mM NaCl, at 1 mL per 10^7^ cells) on ice for 30 min. Tissues were lysed by homogenisation in RIPA lysis buffer (50 mM Tris, 150 mM NaCl, 1% *v*/*v* Triton X100, 0.1% *w*/*v* sodium dodecyl sulphate, 0.5% sodium deoxycholate, 1 mM EDTA) at approximately 1.7 mL per 50–100 mg of tissue. Lysates were supplemented with protease (Complete^TM^ EDTA-free, Roche, Basel, Switzerland) and phosphatase (PhosSTOP^TM^, Roche, Basel Switzerland) inhibitors. Protein concentration was quantified (Bradford protein assay kit, Thermo Fischer Scientific, USA), relative to BSA standards.

Immunoprecipitation from lysates was performed by adding 3 µg/mL of anti-human ADAM10 mouse monoclonal antibody 4A11 [25] and 20 µL of 50% slurry of Protein A/G Sepharose beads (Thermo Fischer Scientific, USA), and incubated overnight at 4 °C on a rotating wheel. The Sepharose samples were washed with lysis buffer and eluted by the addition of 50 µL of SDS loading buffer. The eluted proteins were transferred to fresh Eppendorf tubes, and denatured with 1 µL of 1M dithiothreitol for 3–5 min at 95 °C.

Gel electrophoresis was performed using Bio-Rad 4–15% Criterion™ TGX Stain-Free™ Protein Gels (18 or 26 wells) (Bio-Rad, Hercules, CA, USA) and protein transferred onto Polyvinylidene Difluoride (PVDF) membranes, using the Bio-Rad Criterion™ Blotter. Membranes were blocked in 2% BSA in Tris-buffered saline (TBS), before incubation with primary (overnight at 4 °C) and secondary antibodies (1 h) in BSA/TBS, washed in TBS/Tween (0.1%), and developed with Clarity Western ECL Substrate reagent (Bio-Rad). Blotting antibodies: ADAM10 ab39177 from Abcam (Cambridge, UK), Notch1 (clone D1E11), and Notch2 (clone D76A6) from Cell Signalling Technologies (Danvers, MA, USA), and pan-actin (ACTN05 (C4), Thermo Fisher Scientific, Waltham, MA, USA).

### 4.5. Immunohistochemistry (IHC) and Microscopy

Mouse tumour samples were taken using mid-size (approximately 300 mm^3^) tumours, frozen in O.C.T. compound (Tissue-Tek, Torrance, CA, USA) in cryomolds (Tissue-Tek, USA). Sections (6 µm) from frozen blocks were cut and placed onto electrostatic glass slides, using a cryostat. For IHC, slides were probed with the primary antibody (anti-mouse CD31 (550274), BD Biosciences, Franklin Lakes, NJ, USA) overnight at 4 °C, followed by incubation with the biotinylated anti-rat secondary antibody (Vector Laboratories, Newark, CA, USA, BA-4001-.5), washing 3X after each incubation with TBST. Slides were developed with DAB reagent (3, 3-diaminobenzidine) (Abcam, Cambridge, MA, USA), rinsed in running water, and counterstained for 30–60 s with pre-filtered Mayer’s haemoxylin (Amber Scientific, Largs North, SA, Australia). Slides were then rinsed with running water for 1 min, incubated in Scott’s water solution (Amber Scientific, Australia) for 1 min, and rinsed in running water for 1 min. Slides were dehydrated for 5 min in 70% ethanol, twice for 5 min in 100% ethanol, and twice for 5 min each in xylene. Cover slips were then added to the sections, using DPX mounting solution (Merck Millipore). Stained sections were scanned using the Aperio Scanscope XT (Leica Biosystems, Waverley, VIC, Australia), and biomarker analysis was conducted using Aperio image analysis Positive Pixel Count v9. Alcian blue staining was with 1% Alcian blue in 0.1 N HCL for 30 min, before rinsing 3X in 0.1 N HCL and then distilled water, and imaging with standard light microscopy.

### 4.6. Second Harmonic Generation (SHG) Imaging

Second harmonic generation (SHG) imaging of collagen fibrils in 6-μm thick frozen sections was performed using a Zeiss LSM 980 multi-photon confocal microscope, with the MP laser set at 920 nm, and emission gate at 454–464 nm. A total of 20 slices were taken for each Z stack. SHG signal intensities (maximum grey values) were determined by image analysis using Fiji (ImageJ) software, version 2.9.0, and the CZI-SHG-analysis.ijm script (https://github.com/jrwconway123/SHG-analysis, accessed on 8 May 2025), using the mean of 4 fields of view.

### 4.7. MSC Differentiation Assay

Differentiation assays were using mesenchymal stromal cells (MSCs) derived from B57/Bl6 mouse aorta and grown in MSC growth medium [66]. A total of 8 × 10^4^ MSCs in 5 µL were placed centrally in wells of a 12-well plate, surrounded by a ring of MSC medium, for 2 h, before adding 3 mL of conditioned medium from Wt or ADAM10 KO U251 cells to triplicate wells. Fresh MSC medium and StemPro Chondrocyte Differentiation Basal Medium (Thermo Fisher Scientific) served as negative and positive controls, respectively. The MSCs were incubated for 2 weeks, with media changes twice/week, then fixed in 4% formaldehyde (30 min), stained with Alcian blue solution (pH 2.5, 1% Alcian blue in 0.1 N HCL for 30 min), and washed 3X in 0.1 N HCL and then water, before imaging by standard light microsopy. Staining (background-subtracted signal intensity) was quantified using ImageJ software.

### 4.8. Tumour Xenograft Models

Immuno-deficient NSG mice (Nod/SCID/gamma) (5–6 weeks old) were obtained from the Animal Resources Centre (Canning Vale, Western Australia, Australia). All animals were handled in strict accordance with good animal practice, as defined by the National Health and Medical Research Council (Australia) Code of Practice for the Care and Use of Animals for Experimental Purposes, and approved by the Austin Hospital Animal Ethics Committee. Mice were injected with 5 million cells in PBS subcutaneously on the flank, in groups of six mice (Wt or ADAM10 KO cells). Tumour volumes were determined by calliper measurement (volume = length × width^2^/2), twice weekly. Mice were humanely euthanised at or before the ethical endpoint (tumour volume of 1000 mm^3^), and tumours were recovered for analysis.

## 5. Conclusions

In conclusion, our experiments show the wide effects of ADAM10 KO on protein shedding and expression in human GBM and CRC cells grown in vitro, and the inhibition of tumour xenografts in mice. We identified known and novel ADAM10-regulated signalling mechanisms, with prominent effects being associated with the tumour microenvironment, including blood vessels, ECM organisation, and differentiation, and the expression of the associated protein signatures also correlated with ADAM10 expression in human tumours. Future work incorporating both orthotopic and immunocompetent mouse models will be needed to further investigate the tumour-promoting roles of ADAM10 and its potential as a therapeutic target.

## Data Availability

All data generated or analysed during this study are included in this published article and its Supplementary Information files, and can otherwise be provided by the corresponding author upon reasonable request.

## Supplementary Materials

The following supporting information can be downloaded at: https://www.mdpi.com/article/10.3390/ijms262110684/s1.
