# Supplementary material for: ADAM10 Knockout from Human Glioblastoma and Colon Cancer Cells Modulates Diverse Signalling Networks and Inhibits Tumour Growth In Vivo
Source: Int J Mol Sci. 2025 Nov 3;26(21):10684. doi: 10.3390/ijms262110684 (PMC12608950; doi:10.3390/ijms262110684)
Supplement: Supplementary file 1 [file ijms-26-10684-s001.zip › ijms-3894237v2, suppl. figures.pdf]

Figure S1

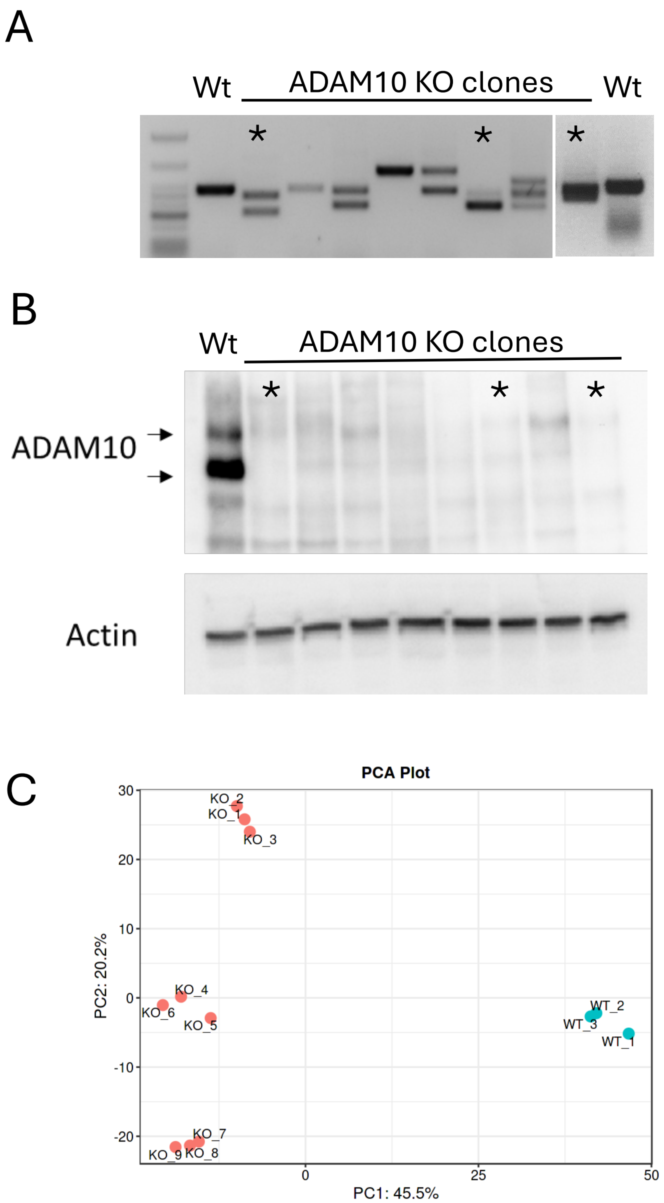

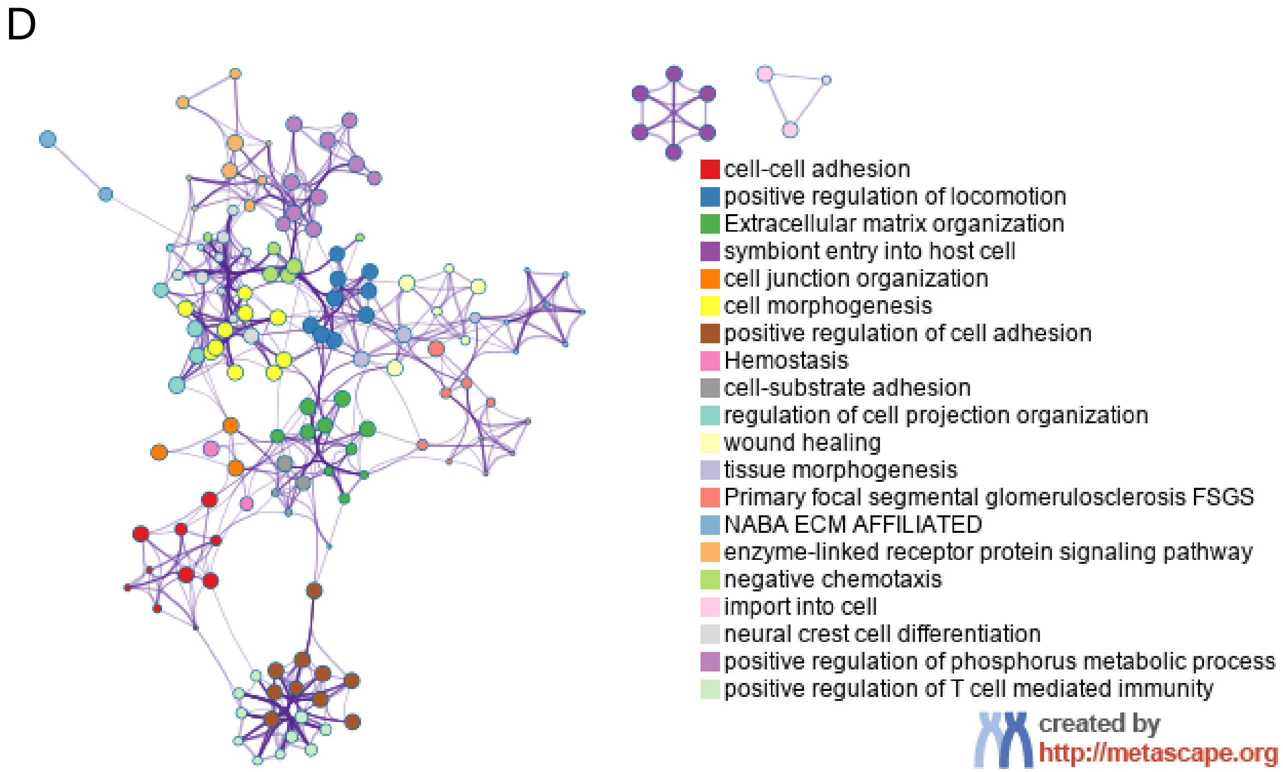

Figure S1. A. PCR screen of genomic DNA from ADAM10 CRISPR clones (*clones used for proteomic analysis). B. Western blot of anti-ADAM10 immunoprecipitates from wild type (Wt) U251 cells or ADAM10 knockout (KO) clones, probed with anti-ADAM10 antibodies (*clones used for proteomic analysis). C. PCA plot of proteomes detected in conditioned media from Wt and three KO U251 clones. D. Network of enriched terms for down-regulated proteins (from Figure 1) colored by cluster ID, where nodes that share the same cluster ID are typically close to each other (Cytoscape.org).

Figure S2A

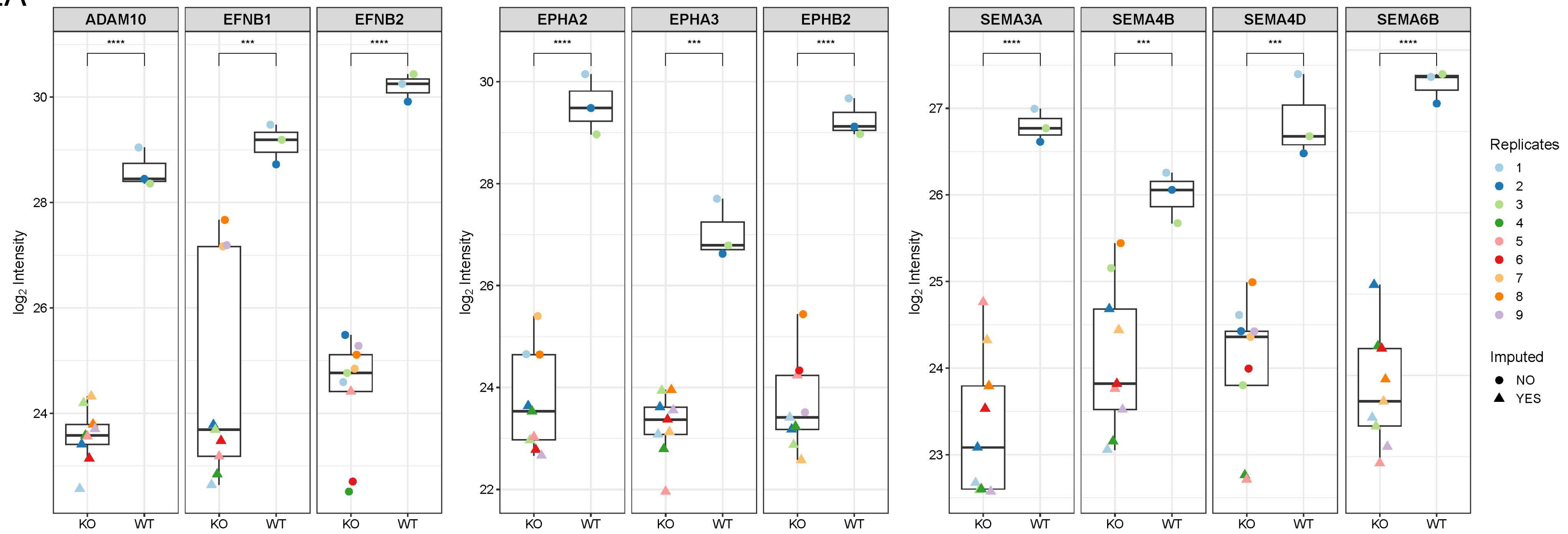

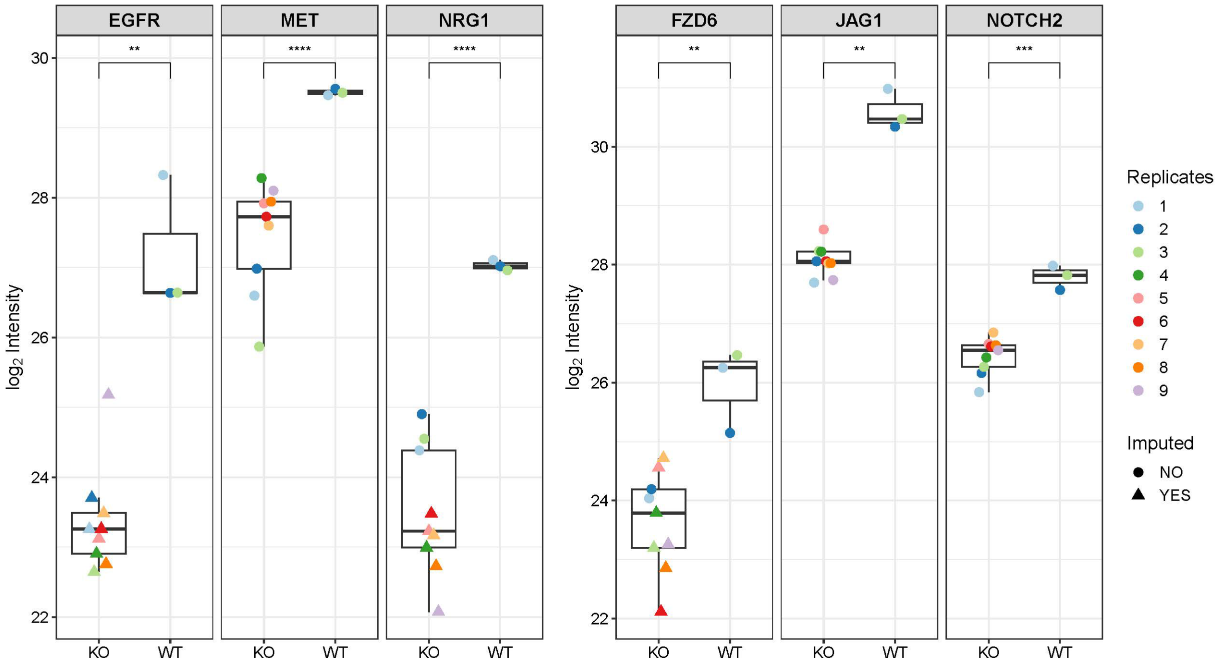

Figure S2A and S2B
Comparison of expression of some key proteins in conditioned medium from U251 Wt and ADAM10 KO cell lines, determined by quantitative mass spectrometry

Figure S2B

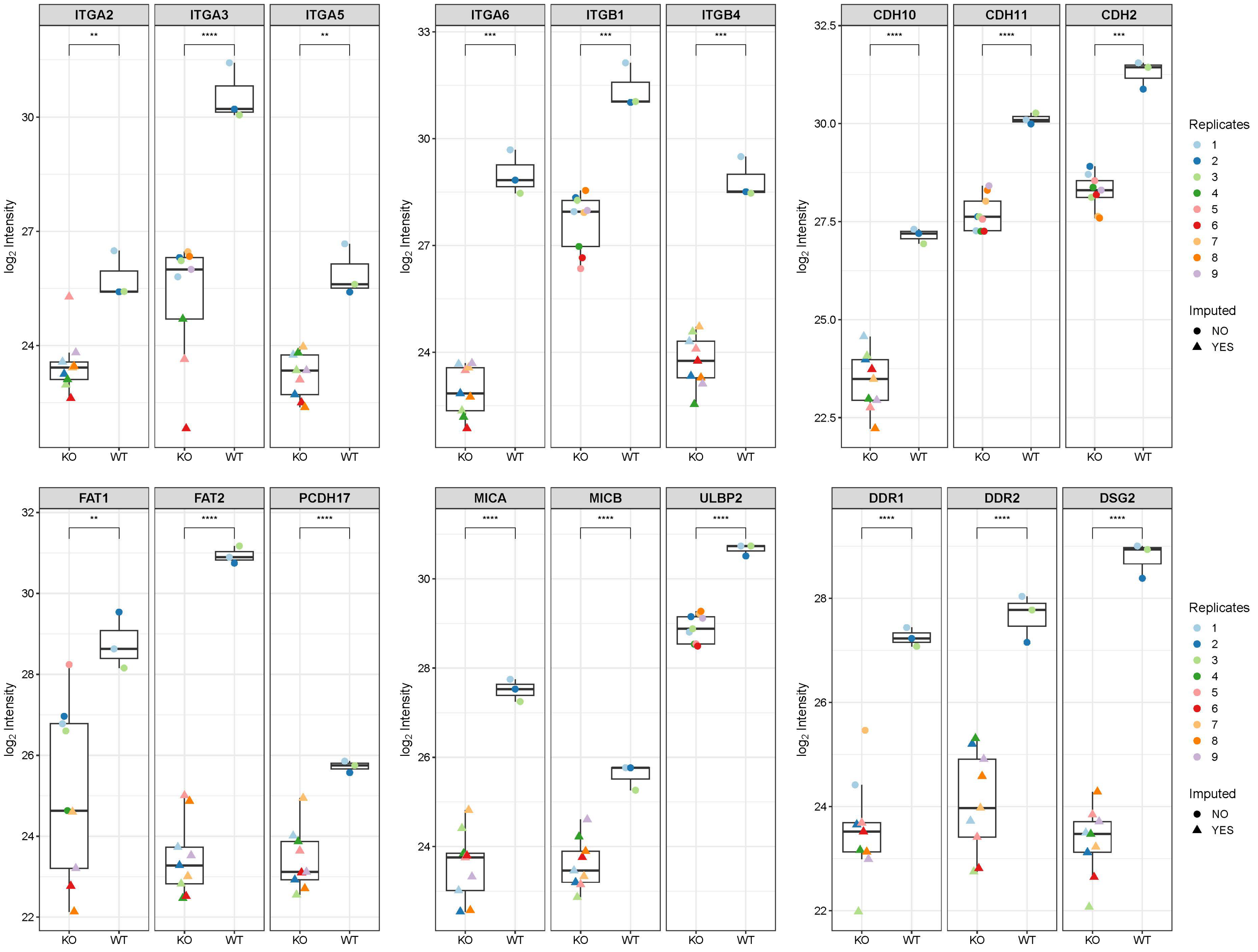
ITGA2
ITGA3
ITGA5
ITGA6
ITGB1
ITGB4
CDH10
CDH11
CDH2
FAT1
FAT2
PCDH17
MICA
MICB
ULBP2
DDR1
DDR2
DSG2
log2 Intensity
KO
WT
Replicates
1
2
3
4
5
6
7
8
9
Imputed
NO
YES

Figure S3

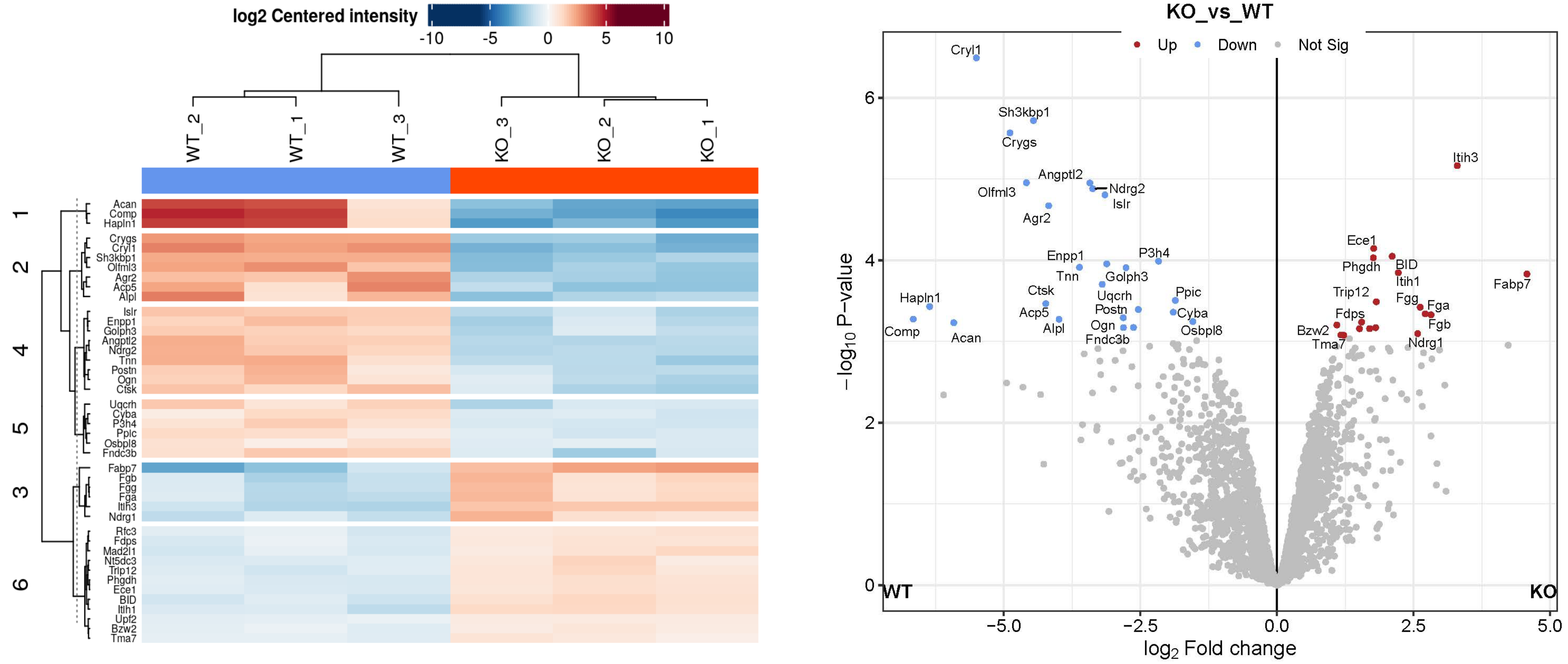

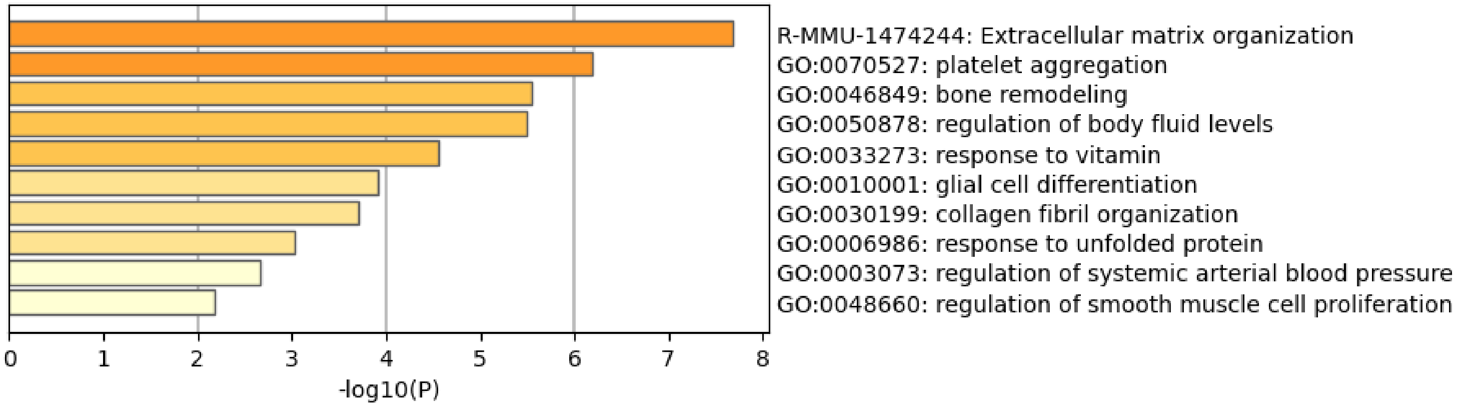

Figure S3. Proteomics analysis of proteins in lysates from Wt and ADAM10 KO U251 tumours, searched against the mouse protein database. A,B. Heatmap (A), and volcano plot (B) of all significantly differentially expressed proteins (≥2-fold). (Blue: down-regulated). C. Gene annotation analysis of significantly differentially expressed mouse proteins: bar graph shows enriched terms across input gene lists, colored by p-values (Metascape.com).

Figure S4

Figure S4. Shared significant protein changes in ADAM10 KO conditioned medium v cells v tumour

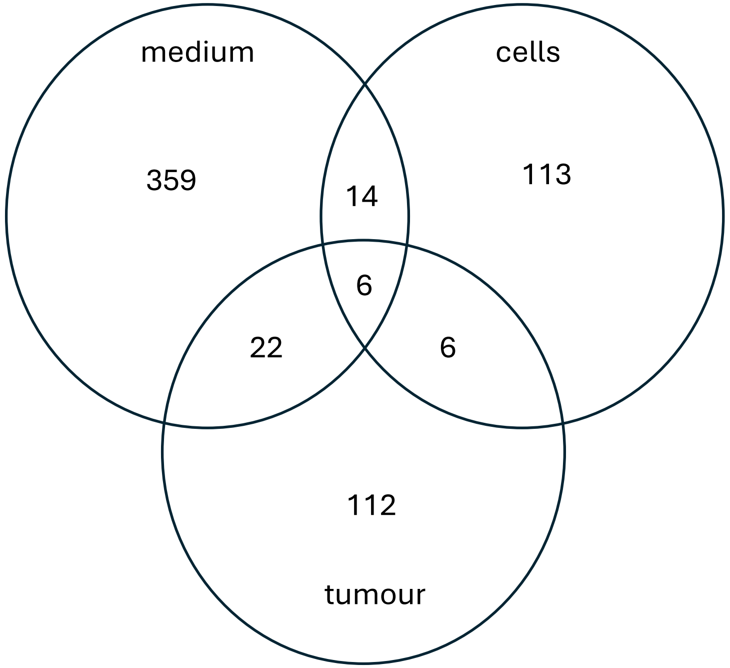

Figure S5

Human

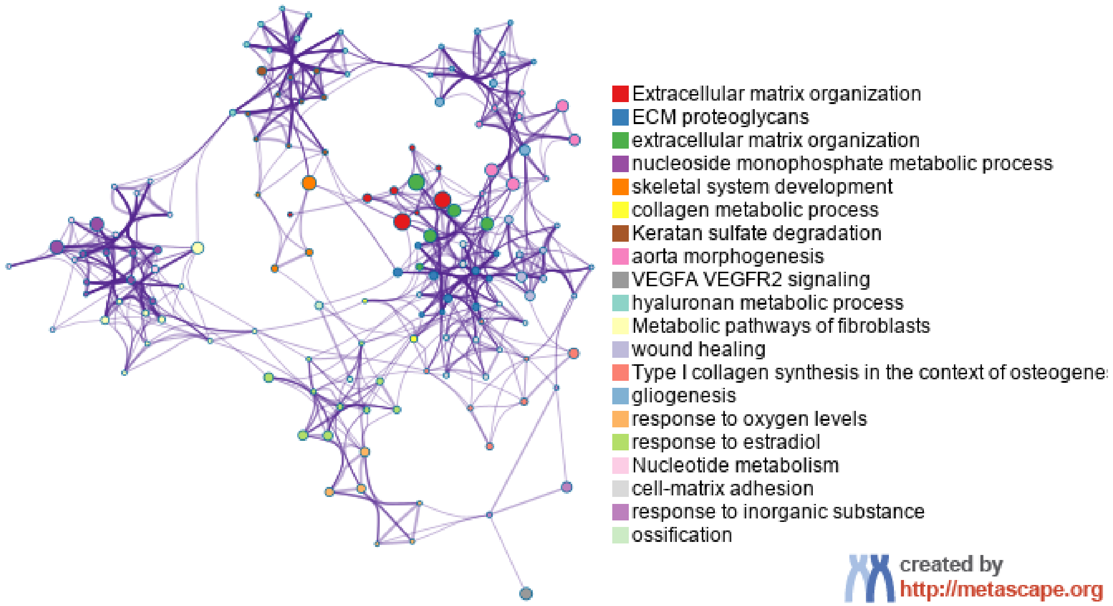

Mouse

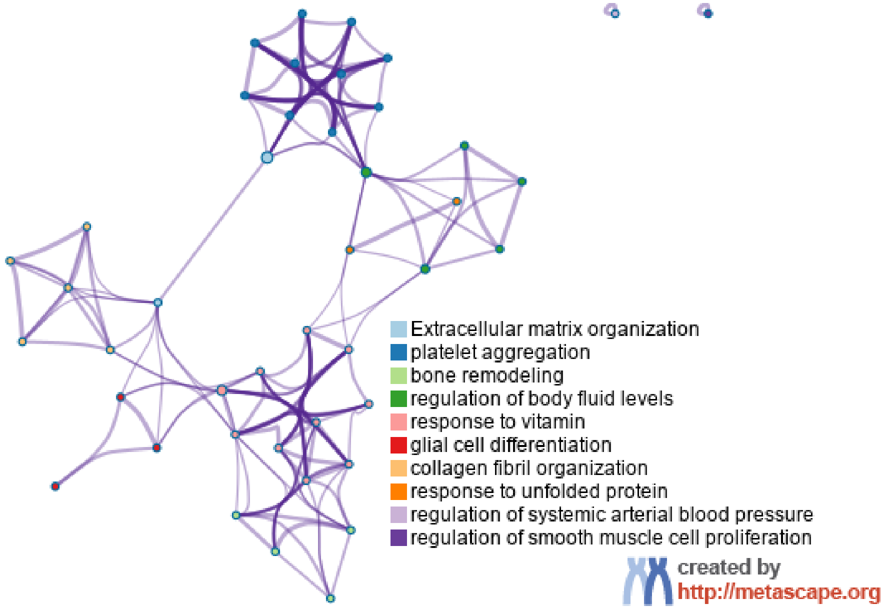

Figure S5. Network of enriched terms for down-regulated proteins in tumours search against human or mouse protein databases (Figure 5, S3) colored by cluster ID, where nodes that share the same cluster ID are typically close to each other (Cytoscape.org).

Figure S6

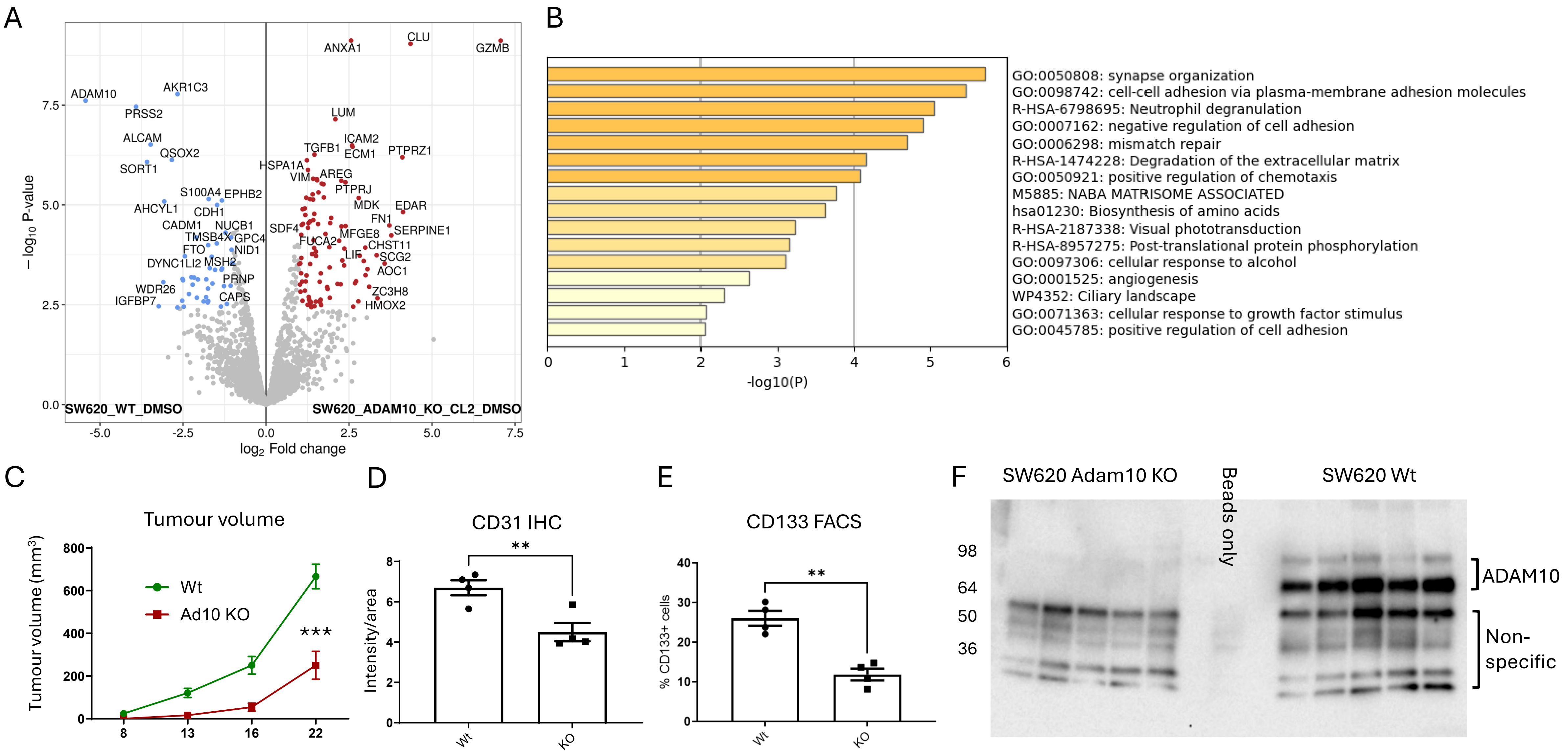

Figure S6. ADAM10KO SW620 model. A volcano plot showing significantly up (red) and down (blue) regulated proteins in conditioned media from ADAM10 KO SW620 cells compared to Wt cells. B. Protein annotation of down-regulated proteins showing enriched terms, colored by p-values (Metascape.com). C. Comparison of Wt and ADAM10 KO SW620 xenograft growth in mice (Mean volume ± SEM, n = 5/group). D. Quantification of CD31 staining of whole tumour sections by IHC. E. Quantification of CD133 staining (% cells) of dissociated tumours by flow cytometry. F. Western blot of anti-human ADAM10 immunoprecipitates from tumour lysates, probed with antibodies for ADAM10. Beads only control: Protein A/G sepharose beads were incubated with Wt tumour lysate in the absence of anti-human ADAM10 antibody. ***$p < 0.001$; **$p < 0.01$

Figure S7

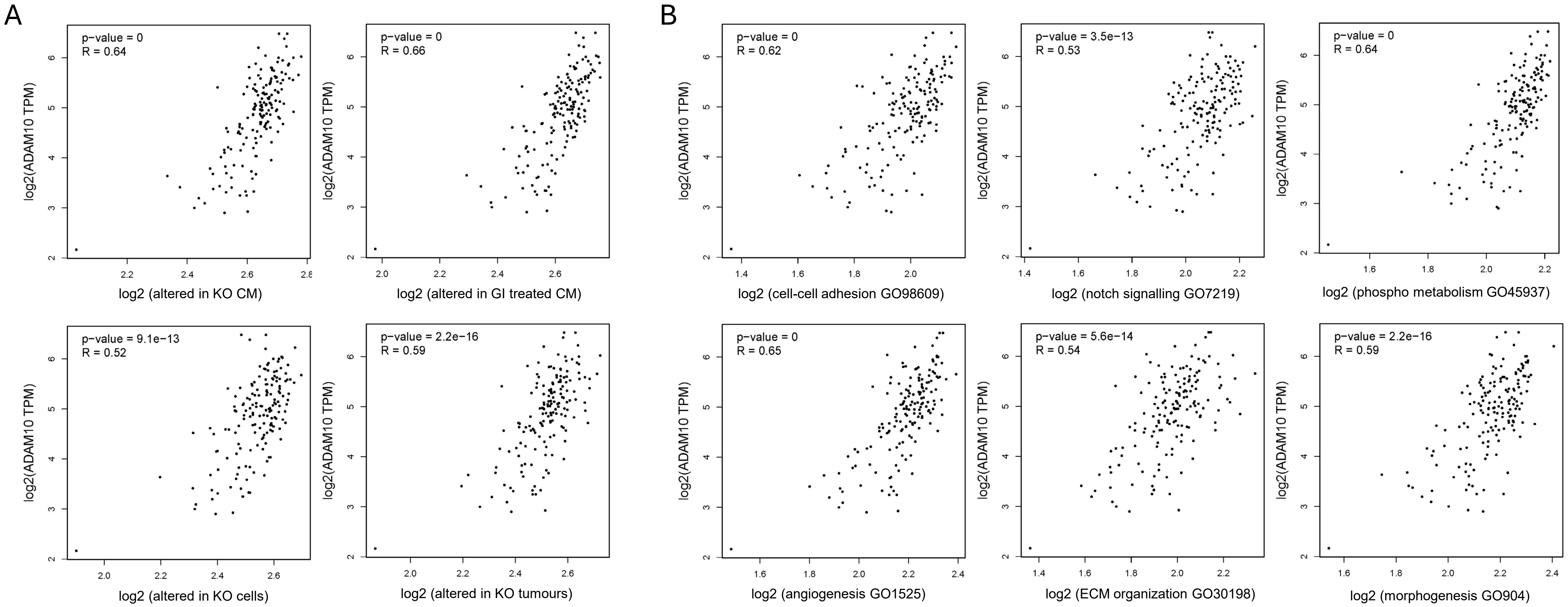

Figure S7. (A) Correlation plots of ADAM10 expression (*y*-axes) in human GBM (TCGA dataset) with expression of ADAM10-regulated protein sets (*x*-axes) identified in U251 models. (B) Correlation plots of ADAM10 expression in human GBM with expression of key GO functional terms identified as enriched in ADAM10-modulated protein sets. TCGA data (http://gepia2.cancer-pku.cn/#correlation).

Figure S8

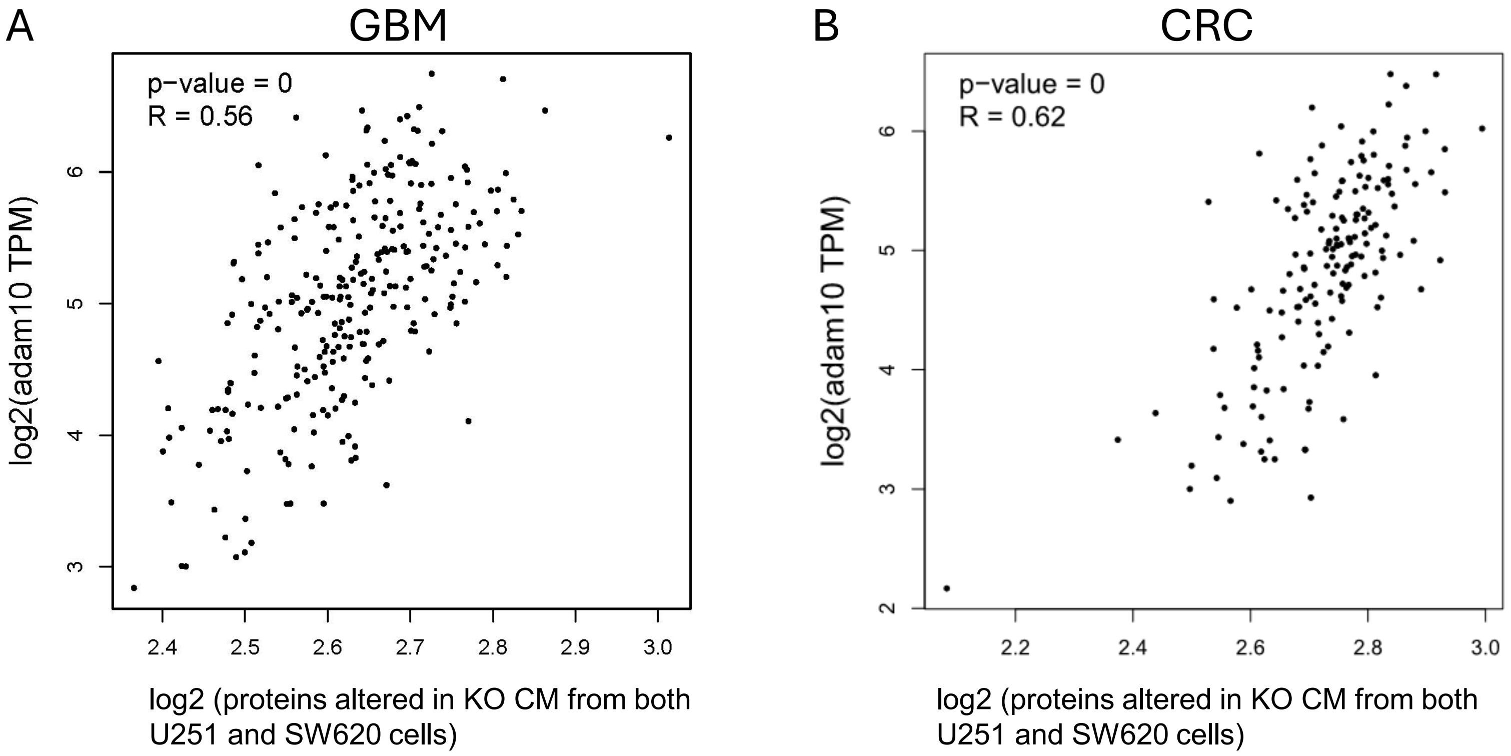

Figure S8. (A) Correlation plots of ADAM10 expression (*y*-axes) in human GBM (TCGA dataset) with expression of ADAM10-regulated protein sets (*x*-axes) identified in secreteome from both U251 GBM and SW620 CRC cells. (B) Correlation plots of ADAM10 expression (*y*-axes) in human CRC (TCGA dataset) with expression of ADAM10-regulated protein sets (*x*-axes) identified in secreteome from both U251 GBM and SW620 CRC cells. (http://gepia2.cancer-pku.cn/#correlation).
